# A randomized double-blind comparison of the double-space technique versus the single-space technique in combined spinal-epidural anesthesia for cesarean section

**DOI:** 10.1186/s12871-020-0948-7

**Published:** 2020-01-30

**Authors:** Eun Hee Chun, Sooyoung Cho, Jae Hee Woo, Youn Jin Kim

**Affiliations:** 1 Department of Anesthesiology and Pain Medicine, Kangnam Sacred Heart Hospital, Hallym University College of Medicine, Seoul, Republic of Korea; 2 Department of Anesthesiology and Pain Medicine, College of Medicine, Ewha Womans University, 25 Magokdong-ro 2-gil, Gangseo-gu, Seoul, Republic of Korea 07084

**Keywords:** Cesarean section, Combined spinal-epidural technique, Obstetric anesthesia, Patient satisfaction, Regional anesthesia

## Abstract

**Background:**

Combined spinal-epidural anesthesia (CSEA) can be performed with either a single-space technique or a double-space technique for cesarean section. We performed a double-blind randomized controlled study to compare the effect of the double-space technique with that of the single-space technique on sensory block level and side effects.

**Methods:**

Parturients undergoing elective cesarean section under regional anesthesia were randomized to receive CSEA with either the double-space technique (double group, *n* = 20) or the single-space technique (single group, n = 20). In the double group, an epidural catheter was inserted at the L1–2 interspace, and dural puncture was performed at the L3–4 interspace. In the single group, the procedure was performed at the L3–4 interspace using the needle-through-needle technique.

**Results:**

There were no differences in time to readiness or intraoperative level of sensory block between the two groups. The postoperative sensory level was maintained at a higher level in the double group than in the single group (1 h postoperatively, *P* = 0.029; 6 h postoperatively, *P* = 0.016). There was no difference between the two groups in terms of side effects. The parturient satisfaction scores 48 h postoperatively were significantly different between groups (9.5 in the double group vs. 8 in the single group, *P* = 0.004).

**Conclusions:**

We conclude that there were no differences in intraoperative variables between the double-space technique and the single-space technique for CSEA. However, double-space CSEA for cesarean section may be beneficial for controlling postoperative pain and improving parturient satisfaction.

**Trial registration:**

The study was retrospectively registered at https://cris.nih.go.kr under the trial ID KCT0002514. Date of registration: October 27, 2017.

## Background

Combined spinal-epidural anesthesia (CSEA) has advantages that compensate for the shortcomings of spinal or epidural anesthesia alone for cesarean section. It combines the best features of spinal anesthesia (rapid onset, intense blockade, and a decreased drug requirement) and epidural anesthesia (titratable anesthesia levels, the ability to extend duration, postoperative analgesia supplementation), and it avoids the disadvantages of each, including different levels between individuals in spinal anesthesia and incomplete motor blocks and missed segments in epidural anesthesia.

CSEA can be performed using either the single- or double-space technique; the double-space technique was introduced first. Brownridge’s first report of CSEA in obstetric anesthesia in 1981 described epidural catheter placement at L1–2 followed by a subarachnoid block at L3–4 [1]. The needle-through-needle technique was described independently by Coates and Mumtaz in 1982 [2]. At that time, the technique was performed with an ordinary epidural needle and a long spinal needle. Currently, a large number of commercial kits are available, designed specifically for needle-through-needle CSEA. The single-space technique, also called the needle-through-needle technique, is the most widely reported CSEA technique in the literature and is likely the most frequently used [3]. However, there is no recent comparison of the two methods, and the method used is chosen according to the anesthesiologist’s preference in clinical settings. In previous studies, the double-space technique had a greater success rate than the single-space technique [4, 5] and a low complication rate [6], but there were also conflicting reports on the success rate [7]. Furthermore, we have found few reports about the comparisons between the two techniques that include block characteristics, side effects, and parturient satisfaction. It is time to think about the meaningful use of the two techniques rather than the kits.

We hypothesized that CSEA with either the single- or double-space technique would make a differences in the sensory block level, the incidence of side effects and the perioperative outcomes.

## Methods

The study was approved in July 2014 by the local Institutional Review Board of Ewha Womans University Hospital, Seoul, Republic of Korea (EUMC 2014–05–032-007) and registered with the Clinical Trial Registry of Korea (https://cris.nih.go.kr) under the trial ID KCT0002514. Written informed consent was obtained from all patients. A prospective randomized, double-blind study was performed at term pregnancy scheduled for elective cesarean section. Parturients with pregnancy-induced hypertension, multiple pregnancies, placenta previa, cardiac diseases, or contraindications to regional anesthesia were excluded. A total of 40 parturients were randomized to receive CSEA with either the double-space technique (double group, *n* = 20) or single-space technique (single group, *n* = 20). The random allocation sequence was created by an anesthesiologist who did not participate in the study using a computer-generated randomization schedule (www.randomization.com). On arrival at the operating room, all parturients were rapidly infused with 10 ml kg^− 1^ of lactated Ringer’s solution. Oxygen was administered at a flow rate of 3 l min^− 1^ through a nasal cannula. Electrocardiogram, noninvasive blood pressure and pulse oximetry monitoring were performed, and baseline values were recorded. After taking the right lateral position, preprocedural ultrasound scanning was performed in a nonsterile manner. Using a 2–5 MHz curved probe (M-TurboTM; SonoSite Canada Inc., Canada), the sacrum was identified first; then, the transducer was moved cephalad, and the intervertebral level was marked with a skin marker. All parturients received local anesthetic infiltration at the L1–2 and L3–4 interspaces using 1% lidocaine prior to CSEA.

In the double group, an 18-gauge Tuohy needle (Perifix®; B. Braun, Melsungen, Germany) was introduced, using a loss of resistance to air to confirm the epidural space. Dural puncture was performed at the L3–4 interspace with a 25-gauge Quincke tip spinal needle (Tae Chang Industrial Co., Ltd., Kongju, Korea). Next, 0.5% hyperbaric bupivacaine 6 mg mixed with fentanyl 25 μg was given intrathecally after the free flow of cerebrospinal fluid was observed. A 20-gauge epidural catheter was inserted through the epidural needle, 3–4 cm into the epidural space. After the Tuohy needle had been removed, the catheter was firmly fixed and covered with gauze so that the level of catheter entry could not be distinguished. For the epidural test dose, 3 ml of 0.375% levobupivacaine with epinephrine (1:200,000) was injected.

In the single group, the procedure was performed at the L3–4 interspace. An 18-gauge Tuohy needle (Espocan®; B. Braun, Melsungen, Germany) was introduced using a loss of resistance to air, and the dura was punctured with a 27-gauge Sprotte needle using the needle-through-needle technique. When free flow of cerebrospinal fluid was observed, 0.5% hyperbaric bupivacaine 6 mg and fentanyl 25 μg were administered. After withdrawal of the spinal needle, a 20-gauge epidural catheter was inserted through the epidural needle, 3–4 cm into the epidural space. The Tuohy needle was removed, and epidural catheter fixation and an epidural test dose injection were performed in the same manner as in the double group.

CSEA was performed by one investigator (YJK). The parturients and the investigator (EHC) were unaware of which group they had been assigned to, and the investigator (EHC) performed all assessments.

The primary outcome measures for this study were time spent on the procedure, time to readiness, and sensory block level. The secondary outcomes included the failure of the block, the incidence of side effects (e.g., hypotension, bradycardia, nausea, dizziness), neonatal outcomes, parturient satisfaction scores (0–10, 0 = unsatisfied, 10 = satisfied; at the end of the procedure, upon arrival at the postanesthesia care unit (PACU), and 48 h postoperatively) and variables associated with postoperative recovery (e.g., pain scores, motor blockade, and sensory level). The total procedure time was defined as the time interval between local infiltration to the skin and the intrathecal injection. Time to readiness was defined as the time from intrathecal injection to the T4 sensory block. The point of intrathecal injection was taken as time 0 min in both groups. Maternal blood pressure was recorded every minute for 10 min and at 5-min intervals for the remaining time. Hypotension was defined as a 20% or greater decrease below the preinduction level or a systolic pressure below 95 mmHg, which was treated immediately with ephedrine 5 mg, i.v., and repeated whenever necessary. Bradycardia (heart rate < 50 bpm) was treated with 0.5 mg of atropine.

At the end of surgery, parturients received an epidural bolus injection: 10 ml of a solution of ropivacaine 0.2% with morphine sulfate 1 mg. Postoperative analgesia was provided with an epidural infusion at 5 ml h^− 1^ of a solution containing ropivacaine 2 mg ml^− 1^ and fentanyl 4 μg ml^− 1^.

The sensory block was tested every minute for 10 min and 1, 6, 12, 24, and 48 h after the operation. The motor block was checked 1 and 6 h after the operation. Sensory levels were checked by cold sensation using an alcohol sponge, and the motor block was assessed using a modified Bromage scale (0 = no block; 1 = weak or absent hip flexion, able to move knees and ankles; 2 = unable to move hips or knees, able to move ankles; and 3 = unable to move any joint). Postoperative pain scores using numeric rating scales (NRS) (0–10; 0 = no pain, 10 = the most severe pain imaginable) were recorded postoperatively at 1, 6, 12, 24, and 48 h.

It was recorded when the patients started urination after foley catheter removal and when the patients observed the first flatus. The time to start walking was also recorded independently.

### Statistical analysis

Statistical comparisons of the continuous variables between the two groups were analyzed with Student’s *t*-test, and sensory and motor block variables were compared using the Mann-Whitney U test. Differences between the two groups in the incidences of side effects such as hypotension, pain, nausea and vomiting and dizziness were analyzed with chi-square tests and Fisher’s exact test when appropriate. A repeated measures analysis of variance was used to test the difference between the two groups in blood pressure. *P* < 0.05 was considered to be statistically significant. SPSS (ver. 18.0, Chicago, IL, USA) was used for the statistical analysis. The data are expressed as numbers, percentages, and medians [range] or means ± standard deviation. From our experience and a previous study [8], using a two-sided design at a significance level of 5% with a power of 80%, an estimated 16 parturients per group were needed to detect a sufficient effect size. Assuming a 20% dropout rate, we designed the study with 20 parturients in each group.

## Results

**Fig. 1:**
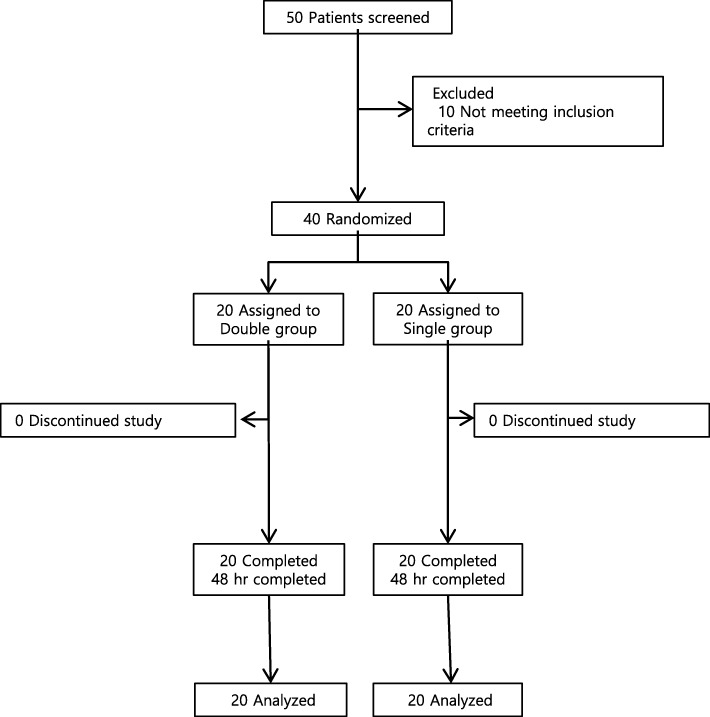
CONSORT chart

Forty parturients scheduled for cesarean sections were enrolled in and analyzed for this study. Figure 1 presents the allocation of parturients into the study groups. No intergroup differences were identified with regard to individual characteristics, duration of surgery, anesthesia time, or total fluid intake and output (Table 1).

**Table 1:** Patient characteristics and clinical features

	Double Group (*n* = 20)	Single Group (*n* = 20)
Age (years)	33.4 ± 2.9	34.0 ± 3.6
Sex (M/F)	0/20	0/20
Height (cm)	159.4 ± 4.6	160.9 ± 4.2
Weight (kg)	67.7 ± 8.6	66.7 ± 7.4
Gestational age (days)	270.8 ± 5.7	265.9 ± 22.7
Operation time (min)	62.5 ± 12.5	55.8 ± 8.5
Anesthesia time (min)	95.0 ± 17.0	87.3 ± 11.9
Fluid intake (ml)	2166 ± 709.3	1990.0 ± 456.7
Output (ml)	1310.0 ± 381.7	1115.0 ± 233.8

Data are presented as the number or mean ± SD. There were no differences between the two groups. Double group = patients who received combined spinal-epidural anesthesia with the double-space technique, Single group = patients who received combined spinal-epidural anesthesia with the single-space technique

*: *P* < 0.05, compared with the single group

Table 2 shows variables associated with the procedures, including procedural time and anesthesia level. There were no differences between the two groups in procedural time. The time from intrathecal injection to T4 sensory block (time to readiness) was 7.5 ± 2.7 min in the single group and 6.6 ± 2.6 min in the double group. The level of sensory block from 1 to 10 min after induction was not different between the two groups. Failure of the block did not occur in either group.

**Table 2:** Variables associated with the procedures

	Double Group (*n* = 20)	Single Group (*n* = 20)	*P* value
Total procedure time (min)	5.0 ± 1.2	5.9 ± 2.4	0.135
Time to readiness (min)	6.6 ± 2.6	7.5 ± 2.7	0.241
Level of sensory block at 1 min	T6 [T3-T11]	T7 [T4-T10]	0.209
Level of sensory block at 3 min	T4 [T2-T9]	T4 [T2-T8]	0.769
Level of sensory block at 5 min	T4 [T1-T7]	T4 [T2-T5]	0.965
Level of sensory block at 10 min	T4 [T1-T5]	T4 [T2-T4]	0.976
Ephedrine dose (mg)	5 [0–30]	10 [0–30]	0.477

Data are presented as the mean ± SD or median values [range]. There were no differences between the two groups: double group = patients who received combined spinal-epidural anesthesia with the double-space technique; single group = patients who received combined spinal-epidural anesthesia with the single-space technique; total procedure time: time interval between local infiltration to skin and the intrathecal injection; time to readiness: time from intrathecal injection to T4 sensory block; the point of intrathecal injection was taken as time 0 min in both groups

**Fig. 2:**
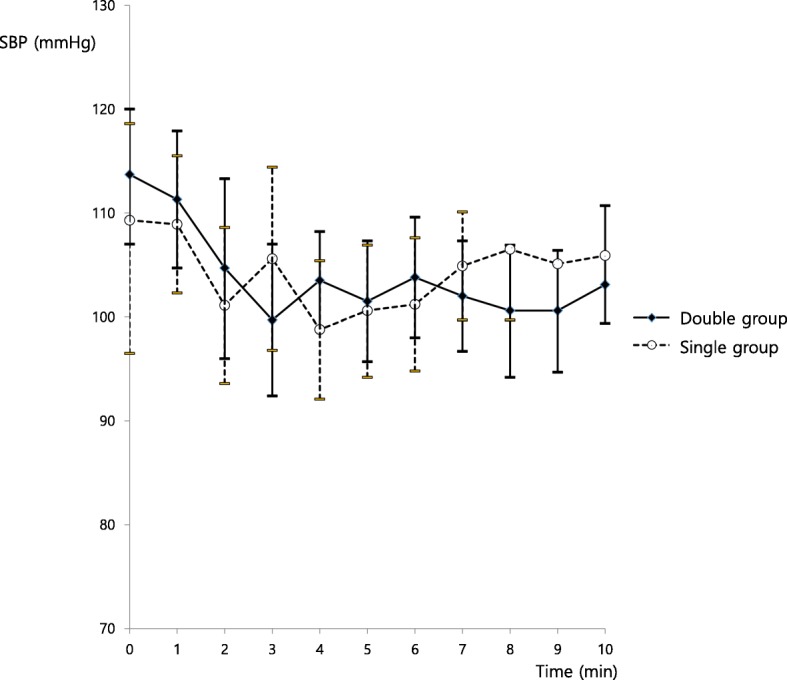
Systolic blood pressure changes after induction. There were no differences between the two groups (*P* = 0.248); SBP = systolic blood pressure; double group = patients who received combined spinal-epidural anesthesia with the double-space technique; single group = patients who received combined spinal-epidural anesthesia with the single-space technique. The point of intrathecal injection was taken as time 0 min for both groups

There were no differences in systolic blood pressure between the two groups during the first 10 min after induction (*P* = 0.248, Fig. 2). There were no differences in Apgar scores between the two groups (Apgar score 1 min: 9.6 ± 0.6 in the double group vs. 9.4 ± 0.8 in the single group; Apgar score 5 min: 9.9 ± 0.3 in the double group vs. 10.0 ± 0.2 in the single group).

During the intraoperative period, the two groups were similar with regard to the occurrence of complications. Hypotension and bradycardia occurred only during the operation (Table 3).

**Table 3:** Incidence of side effects

	Double Group (*n* = 20)	Single Group (*n* = 20)	*P* value
Hypotension	7 (35%)	10 (50%)	0.337
Intraoperative	7	10	
Postoperative	0	0	
Bradycardia	1 (5%)	0 (0%)	1.000
Intraoperative	1	0	
Postoperative	0	0	
Nausea	1 (5%)	3 (15%)	0.605
Intraoperative	0	1	
Postoperative	1	2	
Dizziness	3 (15%)	3 (15%)	1.000
Intraoperative	0	0	
Postoperative	3	3	

Values are numbers (%). There were no differences between the two groups: double group = patients who received combined spinal-epidural anesthesia with the double-space technique; single group = patients who received combined spinal-epidural anesthesia with the single-space technique

The NRS scores for postoperative pain, which were measured at 1, 6, 12, 24, and 48 h, were not different between the groups. The pain scores 12 and 48 h postoperatively showed that pain was well controlled; the NRS scores were 1 in both groups. There were no differences in motor block recovery; however, the sensory block levels of the single group were lower than those of the double group 1 and 6 h postoperatively. The median values were T8 in the double group and T10 in the single group 1 h postoperatively (*P* = 0.029) and T12 in the double group and L1 in the single group 6 h postoperatively (Table 4).

**Table 4:** Variables associated with postoperative recovery

	Double Group (*n* = 20)	Single Group (*n* = 20)	*P* value
Bromage scale 1 h (0–3)	1 [0–2]	1 [0–3]	0.774
Bromage scale 6 h (0–3)	0 [0–1]	0 [0–2]	0.762
Sensory level 1 h	T8 [T4-T11]	T10 [T4-T11]	0.029^*^
Sensory level 6 h	T12 [T6-L1]	L1 [T8-L5]	0.016^*^
Pain 1 h NRS (0–10)	0 [0–2]	0 [0–4]	0.281
Pain 6 h NRS (0–10)	1 [0–3]	0.5 [0–3]	0.300
Pain 12 h NRS (0–10)	1 [0–7]	1 [0–8]	0.801
Pain 24 h NRS (0–10)	1.5 [0–4]	1 [0–7]	0.694
Pain 48 h NRS (0–10)	1 [0–3]	1 [0–7]	0.672
Time required to start ambulation (h)	21.8 ± 4.2	24.1 ± 7.5	0.241
Time required to start urination after foley catheter removal (h)	25.1 ± 4.1	27.4 ± 7.8	0.250
Time required to observe the first flatus (h)	29.8 ± 10.8	32.6 ± 11.3	0.427

Data are presented as median values [range] or means ± SD. NRS; Numeric rating scale, Bromage scale (0 = no block, 1 = weak or absent hip flexion, able to move knees and ankles, 2 = unable to move hips or knees, able to move ankles, 3 = unable to move any joint)

There were no differences between the two groups in postoperative pain score or motor block. The sensory level was significantly higher in the double group than in the single group. ^*^: *P* < 0.05, compared with the single group

The incidence of unilateral leg numbness during epidural patient-controlled analgesia (PCA) infusion was 2 (18%) in the double group and 6 (54%) in the single group, but there was no significant difference (*P* = 0.235).

The time required to start walking independently was 21.8 ± 4.2 h in the double group and 24.1 ± 7.5 h in the single group (*P* = 0.241). The time required to start urination after foley catheter removal was 25.1 ± 4.1 h in the double group and 27.4 ± 7.8 h in the single group (*P* = 0.250). The time required to observe the first flatus was 29.8 ± 10.8 h in the double group and 32.6 ± 11.3 h in the single group (*P* = 0.427). Two parturients in the single group observed the first flatus 2 days after the operation. Mild ileus was observed in their abdominal X-rays, and they recovered without any complications.

The parturient satisfaction scores after procedure completion (satisfaction score OR) and parturient satisfaction scores upon arrival at the PACU (satisfaction score RR) were not different. However, the parturient satisfaction score 48 h postoperatively (satisfaction score 48 h) was higher in the double group than in the single group (9.5 vs. 8, *P* = 0.009, Table 5).

**Table 5:** Parturient satisfaction scores

	Double Group (*n* = 20)	Single Group (*n* = 20)	*P* value
Satisfaction score OR (0–10)	10 [8–10]	10 [8–10]	0.298
Satisfaction score RR (0–10)	10 [7–10]	10 [8–10]	0.089
Satisfaction score 48 h (0–10)	9.5 [8–10]	8 [6–10]	0.009^*^

Data are presented as median values [range]. Satisfaction score OR: the parturient satisfaction score at the end of the procedure; satisfaction score RR: the parturient satisfaction score after arrival at the postanesthesia care unit (PACU); satisfaction score 48 h: the parturient satisfaction score 48 h postoperatively. ^*^: *P* < 0.05, compared with the single group

## Discussion

The present study was a randomized controlled trial comparing two CSEA methods: the single- and double-space techniques after the use of a developed commercial kit for the needle-through-needle technique. The main findings of the present study are that there were no differences in the intraoperative level of sensory block and the incidence of side effects between the two groups. However, the parturient satisfaction score 48 h postoperatively was higher in the double group than in the single group.

Lyons and colleagues reported that separate-needle CSEA had a lower spinal failure rate (4 vs. 16%) and was associated with less hypotension than needle-through-needle CSEA and that the separate-needle group had higher blocks than the needle-through-needle group [9]. However, the anesthesia level during the first 10 min after induction was not different between the two groups, and there were no cases of unsuccessful dural puncture or additional epidural injection during the operation. There were no differences in time from intrathecal injection to T4 sensory block (time to readiness) or systolic blood pressure between the two groups. The main factor determining the intraoperative anesthetic level was the intrathecal injection; furthermore, the site of the indwelling epidural catheter did not make differences in the anesthetic level in this study. In the report of Lyons and colleagues [9], the intrathecal injection drug and parturient position were not described. This point seems to be the cause of the difference in anesthetic level between the present study and Lyons’ study.

A modification of the CSEA technique, for example, epidural volume extension, affects the block height [10]. In the present study, the double-space technique used the L1–2 lumbar interspace, and the single-space technique used the L3–4 interspace for insertion of the epidural catheter. We hypothesized that CSEA with either the single- or double-space technique would affect the sensory block level and side effects; however, there were no significant differences in intraoperative sensory block level and side effects. The mechanism of a higher sensory block with epidural volume extension has been explained as the intrathecal drug being pushed cephalad by the epidural injection [11]. The reasons why augmentation of the sensory block level was not observed in this study were as follows: first, the epidural injection doses were smaller than the epidural volume extension doses. Second, the intrathecal doses were small, so the cephalad spread of the drug was limited.

There were no differences in procedural time between the two groups, and this aspect seems to have little impact on the clinical choice of CSEA technique for anesthesiologists. Casati and colleagues reported that the needle-through-needle technique requires less time, has no greater failure rate, and results in greater parturient satisfaction than the double-space technique [7]. At that time, the development of a spinal needle with a locking mechanism may have contributed to the result. Currently, several commercial kits are commonly used, and the procedural skill in using them has been highly developed. The procedural time depends on the physician’s proficiency rather than on the method.

There was no difference between the groups in the incidences of hypotension, bradycardia, nausea, or dizziness. Although some of these factors are known to affect patient satisfaction [12], there was no difference between the two groups in this study. Therefore, these side effects are not considered to have caused the difference in satisfaction scores between the groups.

We attempted to find aspects to improve maternal satisfaction 48 h postoperatively by reviewing the subjective and objective outcomes. We did not use questionnaires because there is no widely accepted questionnaire for parturients undergoing cesarean section. Some questionnaires are based on subjective discomfort; these were not suitable for this study, which compared the features of two anesthetic techniques [12, 13]. Among the factors affecting maternal satisfaction in previous studies [12, 13], several were investigated in the present study, including procedural time, nausea, dizziness, postoperative pain, motor blockade, time required to start waking independently, time required to start urination after foley catheter removal, time required to observe the first flatus, and unilateral leg numbness. In a retrospective study of labor epidural analgesia, the clinical determinants associated with parturient dissatisfaction were headache, backache, urinary retention, and neural deficit [14]. There were statistically insignificant differences between the two groups for these factors. Furthermore, satisfaction is multidimensional and could be influenced by many factors, such as family support, environmental changes, and mood changes [15]. Although analgesic effectiveness contributes to satisfaction, it is not the only contributor [16]. Among the investigated factors, only unilateral leg numbness developed during postoperative epidural PCA infusion. Therefore, it is presumed to be the cause of the difference in satisfaction scores 48 h postoperatively.

It has been shown that the double-space technique is more advantageous for postoperative pain control. The double group did not show a more profound motor block than the single group; nevertheless, the postoperative analgesic level was higher in this group. CSEA offers advantages, including the ability to eliminate a motor blockade and to achieve a highly selective sensory blockade and optimize analgesia [17]. Our comparison of postoperative pain scores (NRS) did not show a significant difference because the epidural bolus, 10 ml of a solution containing 0.2% ropivacaine and morphine sulfate 1 mg injected in the recovery room, was so effective. Postcesarean pain has at least two components: somatic pain and visceral pain. These are transmitted via T10-L1 spinal nerves [18]. In this respect, the double-space technique is more useful for achieving appropriate postoperative analgesia levels than the single-space technique.

The enhanced recovery after surgery (ERAS) concept has been widely adopted, and there has been great interest in early recovery after a cesarean section [19]. The important aspects of patient recovery were investigated, including the time required to start walking independently, the time required to start urination after foley catheter removal, and the time required to observe the first flatus. These times were shorter in the double group than in the single group; however, the difference was not significant. The factors that affect the time required to start independent ambulation varied. The presence of unilateral leg numbness may affect ambulation. The incidence of unilateral leg numbness during the first 48 h postoperatively was lower in the double group than in the single group, but the difference was not significant. However, the difference between the two groups suggests that the double-space technique may be beneficial for ERAS. Anesthetic considerations for ERAS include postoperative analgesia, fluid management, respiratory function restoration, fasting, and rapid recovery from the motor block. It is believed that there is evidence to support the use of epidurals in ERAS colorectal surgery, but there is no established ERAS guideline for cesarean section. We suggest that these techniques for epidural catheter indwelling can lead to differences in parturient satisfaction and recovery time.

In the present study, the factors affecting parturient recovery were not controlled. Parturients may delay starting ambulation independently without any problems unless they are actively encouraged to walk. This limits the understanding of the anesthetic technique’s effect on a parturient’s recovery. Therefore, additional research is needed to understand the benefits of the double-space technique in ERAS.

## Conclusions

We conclude that there were no differences in intraoperative variables between the double-space technique and single-space technique for CSEA. However, double-space CSEA for cesarean section may be beneficial for controlling postoperative pain and improving parturient satisfaction.

## Data Availability

The datasets analyzed during the current study are available from the corresponding author upon reasonable request.

## Abbreviations

CSEA: Combined spinal-epidural anesthesia
ERAS: Enhanced recovery after surgery
NRS: Numeric rating scales
PACU: Postanesthesia care unit
PCA: Patient-controlled analgesia
